# Interpretable molecular encodings and representations for machine learning tasks

**DOI:** 10.1016/j.csbj.2024.05.035

**Published:** 2024-05-24

**Authors:** Moritz Weckbecker, Aleksandar Anžel, Zewen Yang, Georges Hattab

**Affiliations:** a Center for Artificial Intelligence in Public Health Research, (ZKI-PH), Robert Koch Institute, Nordufer 20, Berlin, 13353, Berlin, Germany; b Department of Mathematics and Computer science Freie Universität, Arnimallee 14, Berlin, 14195, Berlin, Germany

**Keywords:** Explainable, Interpretable, Molecular encoding, Representation, Machine learning

## Abstract

Molecular encodings and their usage in machine learning models have demonstrated significant breakthroughs in biomedical applications, particularly in the classification of peptides and proteins. To this end, we propose a new encoding method: Interpretable Carbon-based Array of Neighborhoods (iCAN). Designed to address machine learning models' need for more structured and less flexible input, it captures the neighborhoods of carbon atoms in a counting array and improves the utility of the resulting encodings for machine learning models. The iCAN method provides interpretable molecular encodings and representations, enabling the comparison of molecular neighborhoods, identification of repeating patterns, and visualization of relevance heat maps for a given data set. When reproducing a large biomedical peptide classification study, it outperforms its predecessor encoding. When extended to proteins, it outperforms a lead structure-based encoding on 71% of the data sets. Our method offers interpretable encodings that can be applied to all organic molecules, including exotic amino acids, cyclic peptides, and larger proteins, making it highly versatile across various domains and data sets. This work establishes a promising new direction for machine learning in peptide and protein classification in biomedicine and healthcare, potentially accelerating advances in drug discovery and disease diagnosis.

## Highlights

• The iCAN encoding method introduces a novel approach to structured input for machine learning models, emphasizing carbon atom neighborhoods for encoding improvement.
• iCAN delivers interpretable molecular representations, enabling the comparison of molecular neighborhoods, pattern identification, and relevance heat map visualization.
• In a large-scale biomedical peptide classification study reproduction, iCAN outperformed its predecessor encoding method.
• When applied to proteins, iCAN surpassed a leading structure-based encoding on 71% of data sets.
• iCAN's versatility extends to all organic molecules, including exotic amino acids and larger proteins.

## Graphical abstract

**Figure:**
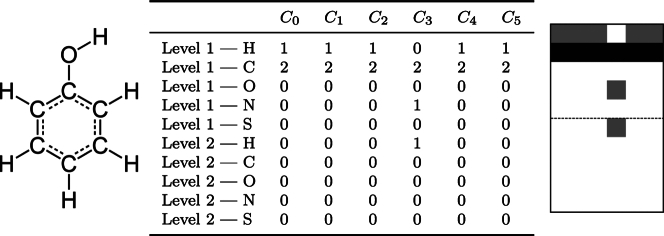

## 1 Introduction

Molecular fingerprinting is a cornerstone of *in silico* molecular studies, virtual screening and machine learning (ML) applications in the field of molecular sciences [1]. Specifically, a molecular fingerprint encodes a molecule by converting its molecular structure into a bit string, which enables the application of various mathematical and computational methods to process, analyze and visually represent the molecule [2]. In the context of ML, encoding a molecule into a machine-readable format allows ML models to effectively capture and learn its inherent chemical structure and properties.

Despite the widespread use of molecular fingerprinting, there is a growing need for alternative encoding methods that can improve the performance of ML models in various molecular science applications. The current vast biomedical space and the limited existence of labeled and balanced data already pose challenges to most ML methods. Moreover, the lack of interpretable molecular encodings hinders the interpretability of models in biomedical research and applications. This poses significant limitations that need to be addressed, particularly in the development of novel interpretable molecular representations for ML, a concept that has not been extensively explored in the existing literature. In response to these changes, we propose an interpretable encoder to advance the field of molecular fingerprinting and its application in various domain applications by demonstrating a new encoding method that improves performance in the ML application domain.

The efficient use of molecular fingerprints relies on the “similar property principle”, i.e., structurally similar molecules tend to have similar properties and similar molecules exert similar biological activities [3], [4], [5]. At its core, the principle implies that molecules with analogous structural features often interact with biological systems or physical environments in comparable ways. For example, molecules with similar shapes or functional groups may bind to the same receptors in biological systems, leading to similar pharmacological effects. Similarly, molecules with comparable chemical compositions may exhibit similar behaviors in environmental processes such as degradation or transport. This principle finds wide-ranging applications, including drug discovery [6], peptide similarity analysis [7], toxicology screening [8], [9], and Quantitative Structure-Activity Relationship (QSAR) modeling [10], [11]. Among their use in a variety of tasks and domains, molecular fingerprints or encodings provide valuable predictive capabilities enabled by ML algorithms trained on labeled data from encoded compounds [12], [7]. For example, knowing whether a peptide can permeate or penetrate the cell membrane.

Two categories of molecular encodings have been investigated so far: sequence-based encodings and structure-based encodings. Sequence-based encodings represent molecules as a sequence of atoms or amino acids, while structure-based encodings represent molecules as three-dimensional structures. The landscape in the literature shows that both sequence- and structure-based encodings are often highly specialized and most often lack interpretability. A large-scale comparative study on peptide encodings by Spänig et al. [13] found QSAR as the only structure-based encoding effective enough for the biomedical classification task. Although there are many sequence-based encodings available, their effectiveness can vary significantly due to factors such as data class imbalance or the method used to create the encoding.

In recent years, there has been an explosion of research into the application of ML, particularly to the prediction of specific molecular properties and structures for amino acid sequences of varying lengths. For instance, molecular embeddings have been proposed to learn molecular representations of peptide and protein sequences. This involves feature learning to identify the best abstraction level for a successful ML task. Deep learning techniques, such as convolutional neural networks, can be used to learn molecular embeddings and obtain protein feature representations by utilizing their sequence representation [14]. This has been made possible by considering different molecular abstractions (*e.g.*, amino acid) and methods (*e.g.*, molecular graph convolutions) which have expanded interest in molecular encodings; some of which inspired researchers to go beyond fingerprints [15]. This is exemplified by models such as AlphaFold and ESMFold, which even attempt to predict tertiary structures [16], [17].

Indeed, an overarching feature of all organic molecules lies in their fundamental composition, centered around carbon atoms as the primary building blocks of the biochemical structure. These molecules typically exhibit a carbon chain forming either a linear backbone or cyclic structures. Extending from this backbone are side chains which bestow diverse biological and chemical properties to the molecules. Drawing upon this universal principle, Hattab et al. [18] devised a parametric fingerprinting method that systematically traverses the carbon chain and captures the multi-level neighborhoods of the carbon atoms. Demonstrating remarkable resource-efficiency, this method achieves accuracy rates comparable to sequence- and structure-based encodings, within the comparative frame of peptide encodings in the aforementioned study. However, unlike existing sequence- and structure-based encodings, it can handle molecules with cycles and unnatural or exotic amino acids, making it widely applicable.

Despite its advantages, the data-driven format in which the atom neighborhoods were recorded hindered its full potential for ML tasks. To tackle this issue, we present the interpretable Carbon-based Array of Neighborhoods (iCAN). Like its predecessor, iCAN adheres to the concept of capturing information about the neighborhoods of the main backbone or the carbon chain. In addition to offering users the choice of only the immediate first neighborhood level, the second level can be added. However, it organizes this information into counting arrays, reminiscent of distribution (frequency) tables, with each column corresponding to a carbon atom in the chain. The encoding array effectively preserves the spatial nature of traversing the carbon backbone, ensuring consistency in the information encoded by each feature. Additionally, the array offers the advantage of visual comprehension, as it can be parsed as a gray scale image. Indeed, the representation of the encoding can be either an array or an image.

Moreover, we offer customizable molecular encodings that afford domain experts the ability to customize the encoding based on their specific considerations for the occurrence of atoms. The user can specify one of three encoding modes based on atomic elements, including the five most common or abundant atoms, *i.e.*, Carbon (C), Nitrogen (N), Oxygen (O), Sulfur (S), and hydrogen (H), the four most common atoms excluding hydrogen, or all atoms present in the data set. The image representation helps users visually compare molecular neighborhoods and identify repeating patterns in the encoding. We introduce an explanatory dimension through the use of relevance heat maps, giving users an intuitive understanding of the selected encoding and its content.

Our method emphasizes the value of both sequence and graph-based information in molecular fingerprinting algorithms, as well as the benefits of visually encoding molecular fingerprints and providing interpretable representations to enhance user insight. By requiring only the chemical sequence of the compound, which may include non-standard amino acids that other encodings are not designed to handle, iCAN extends its applicability to a broader range of domains compared to commonly used encodings.

We demonstrate that our encoding method yields significant performance improvements over its predecessor in the task of property prediction for peptides across 90% of the data. When extended to proteins and dealing with data from previously unexplored domains, iCAN performs robustly. It provides the best encodings for fourteen data sets and outperforms the lead structure-based encoding for 71% of the data. Furthermore, it achieves comparable performance to specialized encodings and even surpasses them in predictive accuracy. We show not only that it outperforms its predecessor, but that it is indeed the most versatile method for encodings with no data set or domain affinities.

In the following sections, we present the results and the methodology of iCAN. Its effectiveness is demonstrated with three experiments which focus on peptide and protein classification. While the first experiment compares iCAN to its predecessor using an extended 62 data sets, the second experiment evaluates iCAN using the golden standard on biomedical peptide classification [13]. The third and last experiment evaluates iCAN on extended domains where other methods fail. This includes but is not limited to data sets with synthetic peptides, unnatural amino acids, *etc.* We evaluate iCAN to showcase its performance and versatility.

## 2 Results

We present our results in four subsections, each covering an important aspect of molecular encodings — their structural differentiation and their interpretability. In particular, while previous work has overlooked the latter, we recognize its importance, especially when ML models are used in molecular science applications. Additionally, as the proposed iCAN builds upon its predecessor the Carbon-based Multi-level Atomic NeiGhborhOod EncodingS (CMANGOES), we further subdivide our results section on the differentiating property of encodings. Specifically, we analyze and compare these two methods, then with other encoding methods from the literature, and later with an extended list of data sets. This characterizes three experiments.

Indeed, to broaden the domain application of our encoding method, we supplemented the data of the golden standard with 12 data sets from various domains, including but not limited to synthetic peptides or foldamers, membrane permeability of cyclic peptides, and toxicity of peptides and proteins, each with different sizes, imbalance ratios, and biomedical properties.

A comprehensive overview of all classification tasks and the corresponding data sets is shown in Table 2. By default, all reported results include both levels of neighborhood information, as our experiments showed that using both first- and second-level neighborhood information leads to better performance in the classification task than using only first-level information.

### 2.1 Experiment 1. Comparison to the baseline

To demonstrate the effectiveness of iCAN as a new alternative in downstream ML tasks and ensure a fair comparison with the Baseline [18], we used the golden standard provided by the PeptideReactor [13] which runs classification via Random Forest classifiers on the encodings and records the corresponding $F_{1}$-score. We adhered to an equivalent configuration with default hyper-parameters in the well-known scikit-learn library [19]. For the data splits, we employ a cross-validation using the RepeatedStratifiedKFold implementation of [19] with a fixed random seed, 5 folds and 10 repeats.

When we considered using iCAN with the third encoding mode, where all atoms present in the data sets, the results show that it outperformed the Baseline across the majority of data sets.

When examining the $F_{1}$-scores for the Baseline and the Alternative, we observed that the $F_{1}$-scores for the Alternative were better. However, the question is whether this is due to chance or whether the $F_{1}$-scores for iCAN are actually higher on average than the Baseline. iCAN yields significant performance improvements over its predecessor in the task of property prediction on the golden standard across 90% of the data. By using the Mann-Whitney U test, we compared the distributions of the $F_{1}$-scores for both methods. iCAN encodings gave a significantly better performance for 54 of the data sets at a 95% confidence. Conversely, it shows a decrease in performance on only three data sets, while no discernible significant difference is observed on the remaining five data sets.

**Fig. 1:**
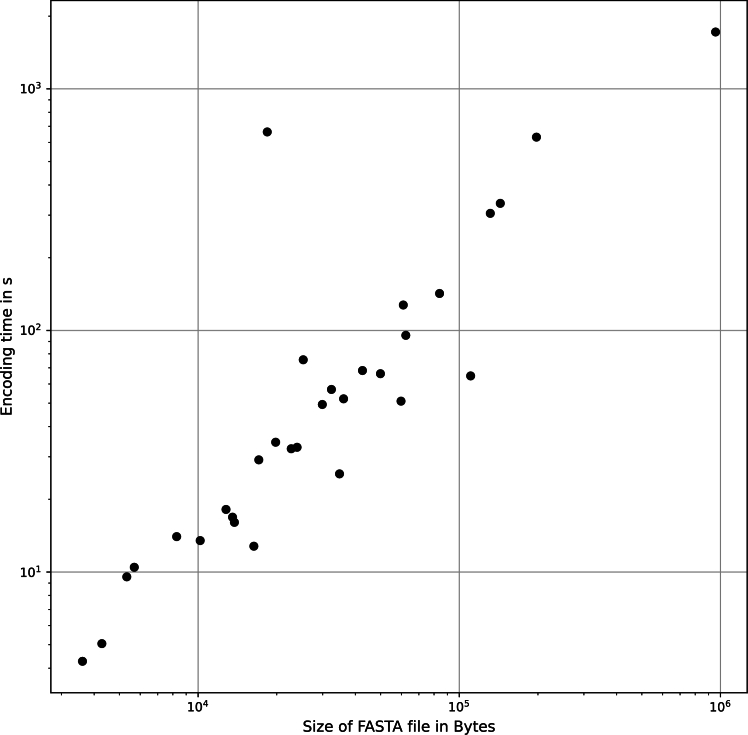
Log-log scatter plot of the encoding time for data sets relative to the size of the original FASTA file. As the file size increases, the encoding time grows linearly. The median encoding time when including first and second level neighborhood information is 1.67 *ms* per byte.

To assess iCAN's resource consumption and scalability, we conducted tests to benchmark the encoding time and the size of the resulting encoded files. In order to ensure reliable measurements, we repeated the encoding process five times and reported the median run time, taking into account any short-term fluctuations in the processing capacity of the machine. The results are presented in Fig. 1. Benchmarking settings are detailed in the supplementary material. We noted that the run time of iCAN increases linearly with the original data set size. On average, iCAN was more efficient in terms of run time than the baseline, taking 1.67 milliseconds (ms) on median to encode 1 byte.

### 2.2 Experiment 2. Comparison to other methods

The proposed encoding method is flexible in terms of the degree of inclusion of atomic neighborhoods and the specific atoms to be taken into account. We evaluated three modes for the elemental alphabet to comprehensively demonstrate the performance differences with respect to other established encoding methods. The first mode included hydrogen, carbon, nitrogen, oxygen, and sulfur. The second mode included carbon, nitrogen, oxygen, and sulfur. The third and last mode included all atoms present in the data set and characterized a data-driven mode.

**Fig. 2:**
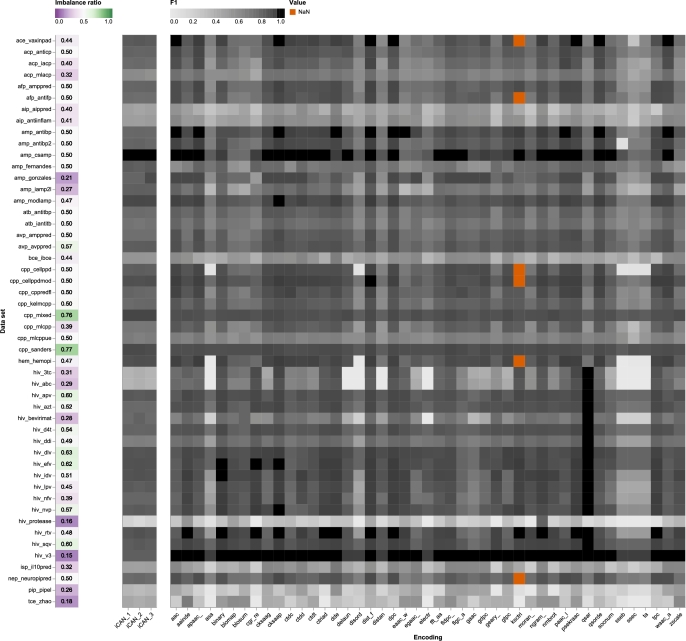
Experiment 2. Heat map comparing *F*_1_-scores for the binary prediction task on three iCAN encodings *versus* 45 state-of-the-art peptide encodings on 50 data sets with different imbalance ratios. All three iCAN encodings use first- and second-level-neighborhood information, for the element alphabet they use the five most abundant elements including hydrogen (iCAN_1), excluding hydrogen (iCAN_2) or collect all elements that appear in the data (iCAN_3).

By systematically exploring different encoding parameters, we gained valuable insight into how different configurations affect performance in the context of binary peptide classification and how iCAN compares to other state-of-the-art encodings. Our results are presented in Fig. 2. iCAN achieves similar performances for all three modes. This is likely due to the fact that the peptides presented here consist only of the twenty standard amino acids and thus there is little difference between the data driven mode 3 and the modes collecting the frequencies of standard elements such as mode 1 and 2. Across all datasets, the performance of iCAN is consistently comparable to the best performing encodings, indicating that its performance achieves state-of-the-art standards but does not manage to overcome the challenges posed by particularly difficult datasets such as hiv_protease. However, it does also perform well on datasets where many encodings fail, such as the particularly unbalanced datasets pip_pipel and tce_zhao iCAN reached the highest $F_{1}$-scores among all 45 encodings of the golden standard on two data sets, *i.e.*, amp_fernandes and cpp_mixed.

### 2.3 Experiment 3. Comparison with extended domains

After having supplemented the data to a total of 62 data sets to include more domain applications, iCAN outperformed the Quantitative Structure-Activity Relationship (QSAR) method on all data sets except the HIV and the *acp_mlcap* data set; accounting for 71% of the data. In addition and as a highlight, the proposed methodology proved to be very robust across the larger and more diverse domain applications range and furthermore the only encoding able to handle all datasets.

Table 1 showcases iCAN as the best encoding for 14 data sets with the highest generalizability and was able to handle unnatural and exotic amino acids and molecules with cycles. These data sets covered the domain applications of antimicrobial peptides, cell penetrating peptides, *β*-peptide foldamers with unnatural amino acids, soluble *E.coli* proteins, protein toxicity, antiviral peptides, amyloidogenic hexapeptide sequences, fungal and oomycete effector proteins. The supplementary materials give a general review of data sets for which iCAN appears in the top 3 encoding methods.

**Table 1:**
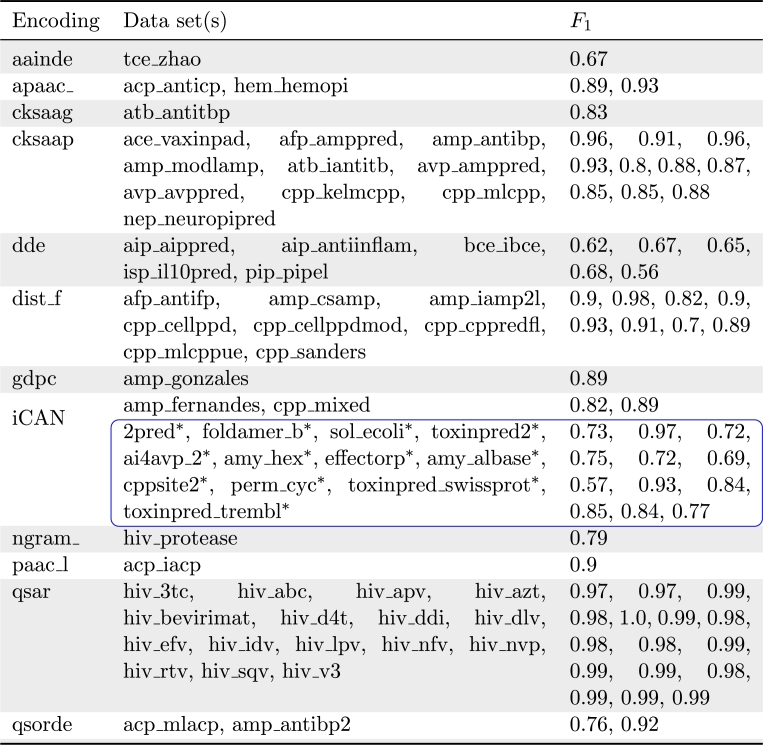
Experiment 3. Overview of the best performing encoding method by alphabetical order. Data sets are aggregated per encoding. A total of 62 data sets is presented. F1 values are rounded. Data sets marked with an asterisk (^⁎^) and encircled in blue could only be encoded using the iCAN method.

### 2.4 Interpretability

**Fig. 3:**
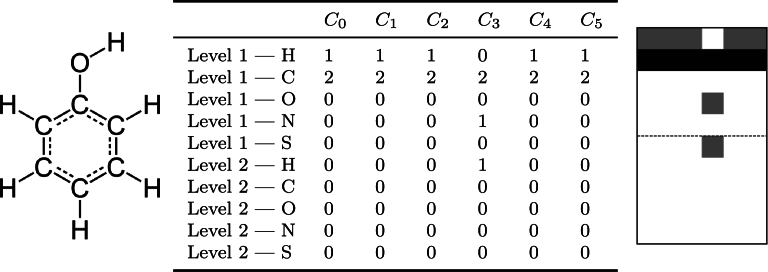
Example array and image representation of the phenol molecule. Left: Phenol or C_6_H_6_O has the SMILES specification: C1=CC=C(C=C1)O. The phenol has a stable conjugated system with a hybrid resonance due to the delocalized or free electrons in the aromatic cycle. Middle: The array of the encoded phenol molecule created by the iCAN method using the first mode, including the hydrogen atoms. The table collects the frequencies of hydrogen, carbon, oxygen, natrium and sulfur in the neighborhoods of all carbon atoms in the molecules backbone. Right: The image representation of the frequency table corresponding to the encoded phenol molecule which may be used for additional interpretability of the encoding through the visual channel.

iCAN facilitated the creation of interpretable molecular encodings and representations. For one, the array and image representations of the resulting encodings are easily exportable, as seen in the example of the phenol molecule in Fig. 3.

The image representations of the molecular encodings are used to easily spot patterns and identify repeating signals in the encodings.

**Fig. 4:**
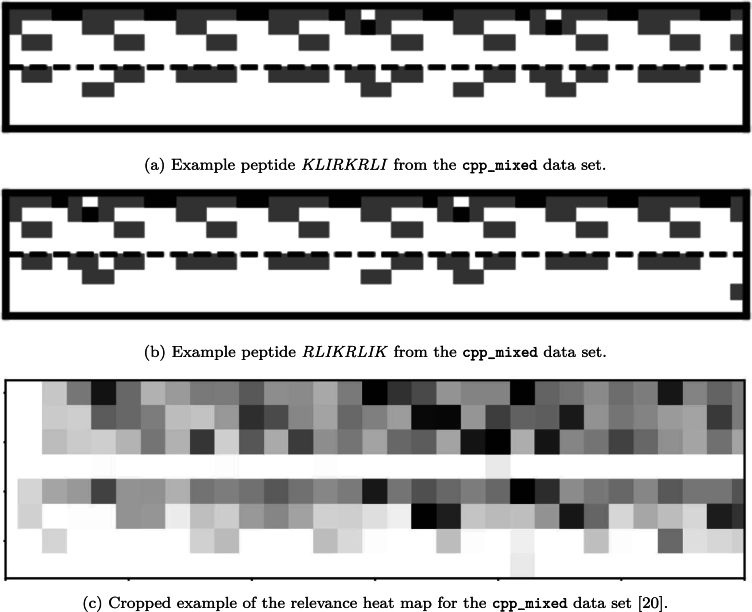
Example interpretable molecular encodings and representations from the cpp_mixed data set. Example image representations of two encoded peptides are shown. Cropped example of the relevance heat map. iCAN was employed using the second mode to create the encodings, excluding the hydrogen atoms.

One such pattern was easily seen in the case of two peptides coming from the cpp_mixed data set. The peptides shown in Fig. 4a and Fig. 4b have similar FASTA representations, a property also reflected in the image representations of their respective encodings. By closely inspecting the figures, we observed a repetition of carbon molecules with almost exactly the same side chains consisting of oxygen and nitrogen atoms. The lack of sulfur atoms on the side chains was another notable observation. Another example relevance heat map is provided in the supplementary material for the ace_vaxinpad data set.

Relevance heat maps, in turn, can be created by graphically mapping measures of feature importance into the image space. One such heat map of the cpp_mixed data set is presented in Fig. 4c. These heat maps visually show which parts of a molecule are relevant to the prediction of a particular property, indicating that these parts are indeed relevant to the presence of that property in the compound. While a recent study used relevance heat maps [21], the relevance of certain parts of molecular fingerprints was determined without linking this relevance back to the molecules. This connection makes it easier for users and domain experts to investigate which molecular parts or molecules play an important role in the ML task.

## 3 Conclusion

The interpretable Carbon-based Array of Neighborhoods (iCAN) presents a versatile method for molecular encodings and representations, applicable to peptides, proteins, and molecules with exotic amino acids and cycles. With its efficient runtime, scaling linearly with dataset size, iCAN proves suitable for large datasets without significant computational overhead. Encodings can be visually interpreted from grayscale images, highlighting molecular similarities. Moreover, iCAN outperforms its predecessor CMANGOES on 54 out of 62 datasets and competes with state-of-the-art methods outperforming all 45 encodings on two data sets. It demonstrates superior generalization across diverse domain applications and accommodates exotic amino acids and cyclic molecules. Notably, iCAN stands as the first sequence-based encoding to compete with structure-based encodings. It preserves the spatial characteristics of molecular data and provides visual comprehension through image representations. Its customizable nature enables users to tailor encoding modes based on atomic elements, widening its applicability across diverse domains. Consequently, researchers in bioinformatics, cheminformatics, and molecular biology can benefit from iCAN's accuracy and interpretability in molecular data analysis. Moreover, the pharmaceutical and biotechnology industries can utilize iCAN in drug discovery and development, leveraging its predictive capabilities for identifying potential candidates and assessing their efficacy and safety. The interpretability feature also enhances regulatory submissions where understanding the model's decision-making process is pivotal.

## 4 Discussion

First, our experiments show that iCAN is very competitive with state-of-the-art encodings, while being resource-efficient and allowing for a wider range of applications, such as molecules with exotic or unnatural amino acids or molecules with cycles.

Second, previous research has suggested that the use of a single encoding of a molecule is inferior to an ensemble of different encodings, each of which captures different aspects of the molecule. Because the resulting encodings and representations capture atomic-level information, we think it would be a good extension to models capturing information that the encoding might ignore, such as physical distance of parts in the molecule or three-dimensional structure. For this purpose, it would be interesting to analyze the correlation between the predictions based on different encodings to determine the potential of ensemble encoding [21]. For example, stereo-isometric information is not currently encoded and may prove useful in various molecular fingerprinting tasks.

Third, a few notes on the use of the two most important parameters of the encodings: Number of Neighborhood Levels and choosing the Element Alphabet. The best choice for the given data sets was the inclusion of first- and second-level neighborhood information. This is likely due to the relatively short length of side chains for peptides, resulting in the information of nearly every atom of an amino acid included in these levels. Preliminary results for compounds with longer side chains indicate that it is advisable to choose all neighborhood levels. This will ensure that most of the atoms are covered by the encoded carbon neighborhoods. With respect to the choice of the element alphabet, there was no clear evidence that the exclusion of the hydrogen atom improves the prediction accuracy of the encodings. In addition, adapting the data-driven alphabet mode has its own advantages and disadvantages. Indeed, including all atoms in the data set increases the amount of information in the encoding and may reasonably improve prediction accuracy. However, there will be problems with the comparison of the encodings of different data sets, which may therefore have used different element alphabets in the encoding. Similarly, the addition of new data points (*i.e.,* organic molecules) to a data set would require verification that no new atoms have been added. If not, a complete re-encoding of the entire data set would be required in order to capture new atoms in the element alphabet.

Fourth, the ability to represent the encoding as a gray scale image opened up the possibility of using the image representation to visually explain the decisions made by a classifier through relevance heat maps. Researchers and users can verify that the model works as intended. The relevance heat map uses the image representation of the encoding to shed a light on which region in the encoding attributed most to the classification decision. Similarly to artificial neural networks, where individual pixels in the input image are identified with the highest activations, it allows us to identify the highest signal contribution. To our knowledge, this is the first time an interpretable dimension has been proposed. This greater level of visual detail is afforded by our interpretable method. On one hand, the image representations can aid the users both in the understanding of the encoding and the comparison of different molecules through the visual medium. On the other hand, the relevance heat map allows the identification of signal contribution at a data set level.

Fifth, the use of a data set-level relevance heat map can help mitigate potential misuse in the design and testing of *in silico* peptides and proteins. Future work may focus on the addition of a peptide- or protein-level relevance heat map, where the visual representation would allow researchers to assess the signal contribution of a peptide or protein. This can be seen as part of a testing process that can help to understand the properties of peptides and proteins and ultimately contribute to the development of more reliable and effective biologically active compounds.

Sixth, the over representation of HIV-specific data sets in Table 2 is important to note. When browsing the classification results of the best encoding per data set in Table 1, the over representation of these data sets leads to the over representation of the QSAR method best suited for the HIV-specific data. Thus, the question arises whether the QSAR method has an affinity for a particular data set or domain. Answering this question was outside the scope of this work but will be the subject of further investigation. However, it is important to note that the associated data sets represent a major challenge for all other sequence- and structure-based encoding.

**Table 2:** Overview of the domain applications and their associated 62 data sets. Explanations are provided for each classification task in the given domain application. A total of 62 data sets are reported.

Domain	Explanation	No. data sets	References
A-cell epitopes	Prediction of peptides for modulating antigen presenting cells (modulating/non modulating).	1	[28]
Anticancer peptides	Prediction of peptides with cytotoxic efficiency against cancer cells (cytotoxic/non-cytotoxic).	3	[29], [30], [31]
Antifungal peptides	Prediction of peptides with anti-fungal efficiency (anti-fungal/not anti-fungal).	2	[32], [33]
Anti-inflammatory peptides	Prediction of therapeutic peptides against inflammatory diseases (anti-inflammatory/not anti-inflammatory).	2	[34], [35]
Antimicrobial peptides	Prediction of peptides with anti-microbial efficiency (antimicrobial/not anti-microbial).	7	[36], [37], [38], [39], [40], [41], [42]
Amyloidogenic peptides	Prediction whether peptides produce amyloid deposits, which may be deposited in organs or tissues under unnatural conditions such as Alzheimer's disease.	2	[43]
Antitubercular peptides	Prediction of peptides with anti-mycobacterial efficiency (antitubercular/not anti-tubercular).	2	[44], [45]
Antiviral peptides	Prediction of peptides with anti-viral efficiency (anti-viral/not anti-viral).	4	[46], [47], [48]
Linear B-cell epitopes	Prediction of B-cell epitopes (B-cell epitope/no B-cell epitope).	1	[49]
Cell-penetrating peptides	Prediction of peptides with penetration capability of cell membranes (cell-penetrating/non cell-penetrating).	10	[20], [50], [51], [52], [53], [54], [55], [56], [57], [58]
*β*-peptide foldamers	Prediction whether peptides are *β*-amino acid oligomers and can adopt stable secondary structures.	1	[59]
Hemolytic peptides	Prediction of peptides with hemolytic susceptibility (susceptible/resistant).	1	[60]
Human Immunodeficiency Virus (HIV)	Prediction with the HIV peptides show drug resistance to various drugs.	17	[61], [62], [63], [64]
Immuno-suppressive peptides	Prediction whether peptides reduce the activation or efficacy of the immune system.	1	[65]
Neuro-peptides	Prediction whether peptides are synthesized and released by neurons.	1	[66]
Permeability of cyclic peptides	Prediction of membrane permeability in cyclic peptides.	1	[67]
Pro-inflammatory inducing peptides	Prediction whether peptides can increase inflammatory reaction as defense against pathogens.	1	[68]
Soluble *E.coli* proteins	Prediction whether an *E.coli* protein is soluble or aggregation-prone.	1	[43]
Linear T-cell epitopes	Prediction whether a peptide is an antigenic determinant, which is recognized by T-cells.	1	[69]
Toxic peptides	Prediction whether peptides are toxic.	2	[70]
Toxic proteins	Prediction whether proteins are toxic.	1	[71]

Seventh and last, while the current methodology focuses on carbon chains for peptides, proteins, and other organics, its ideas can easily be extended to molecules with a backbone of one or more non-carbon elements. For example, polysiloxanes whose backbones consist of alternating silicon-oxygen bonds, or even polyphosphoesters supported by alternating phosphorus-oxygen bonds. Indeed, its proven domain-agnostic nature is a very promising stepping stone.

## 5 Methods

In this section, we elucidate the fundamental principles and algorithm of iCAN, highlighting its encoding core concept. Additionally, we expand upon the experimental framework devised for evaluating the efficacy of iCAN in the context of peptide classification across diverse domains. Furthermore, we provide a comprehensive account of the methodology employed in generating interpretable relevant heat maps, which allow domain-specific experts to leverage these heat maps to systematically explore and discern intrinsic characteristics of the data set, thereby gaining valuable insights.

### 5.1 Encoding creation

The proposed encoding iCAN, like its predecessor, centers on the pivotal role of the carbon chain as the fundamental backbone in numerous organic molecules, notably within proteins and peptides. This property underscores the foundation of our study. Within these carbon frameworks, pivotal connections to side chains exert substantial influence on the inherent properties of the molecules [22]. Distinguishing our work is the innovative representation of neighborhood information through the utilization of a counting array. This approach meticulously preserves the spatial arrangement of the carbon chain augmenting the representation of the molecules. The resulting structural rigidity proves to be advantageous for ML classifiers, facilitating the streamlined identification of intricate patterns that correlate with specific biochemical properties. To effectuate the encoding of the provided data sets, a sequence of operations is carefully followed.

#### 5.1.1 Data preparation

Before starting with the encoding, the algorithm transforms the input data into the correct format, builds a graphical representation, optionally collects all unique elements in the input and collects the connectivity information from the graph in a neighbor directory.

Data format transformation: Initially, the method accommodates a broad spectrum of molecules including proteins, and more broadly, any organic compound (*i.e.*, carbohydrates, lipids or fats, proteins, and nucleic acids) featuring a carbon chain, encompassing cyclic structures and branching chains. The input data can be provided in either the FASTA or SMILES format. However, if the data is in FASTA format, it transforms to SMILES format leveraging the provided function convert_fasta_to_smiles. This conversion step is crucial for subsequent processing and encoding with iCAN.

Conversion to Graph Representation: Upon reception of a molecule, the method leverages the pysmiles package for the conversion process, transforming the molecule into a graph representation. In this graphical depiction, atoms within the molecule are denoted as nodes. Each node is annotated with the elemental abbreviation corresponding to the atom it represents (e.g., C for carbon, H for hydrogen).

Optional Unique Element Collection: The algorithm collects all unique elements that appear in the input data. This is only done if the third mode is used, in which we choose to count the frequencies of all elements which appear in the data.

Atom Identification: In order to ensure a deterministic algorithm and preserve the correct sequential arrangement within the carbon chain, a unique numerical index is assigned to each carbon atom. Consequently, a neighbor directory is established containing the neighboring vertices as a list for each level. This directory serves to encapsulate the connectivity information of each atom within the molecule. Subsequently, another directory is generated based on the neighbor directory. This directory facilitates the conversion of vertices into corresponding elemental representations. Together, these two directories play a pivotal role in maintaining the structural integrity of the molecule during the processing stages.

#### 5.1.2 Configuration and algorithm

In presenting three distinct options for iCAN, the constitution of the Element Alphabet is contingent upon the user's input. This alphabet encompasses the selective elements to be counted for each carbon in the molecules, whose frequencies are documented in the counting array while excluding other types of atoms. Users are afforded the flexibility to choose from three distinct modes:

1. The first mode involves the five most abundant elements found in proteins: hydrogen (H), carbon (C), oxygen (O), sulfur (S), and nitrogen (N).
2. The second mode entails excluding hydrogen and limiting the element alphabet to carbon (C), oxygen (O), sulfur (S), and nitrogen (N).
3. The third mode entails adopting, where all peptides in the data sets are parsed, and all unique atoms are included in the counting array.

The most common elements in organic compounds are hydrogen, carbon, oxygen, nitrogen, and sulfur, with first four elements being the most prevalent [23].

Furthermore, following the choice of the selective elements mode, it is imperative to define the *neighboring level*, denoting the extent to which neighboring elements linked to carbon atoms will be taken into account. This configuration determines the depth of information integrated around the carbon atoms.

Post-configuration, the algorithm systematically gathers information regarding the carbon neighborhoods based on the specified mode or the *element alphabet* and *neighboring level*. Starting from the graph created in the data preparation step, the carbon chain is sequentially traversed. In the case of a branching in the backbone, one path is followed first to its end, then the second path is traversed from the point of the branching. Cycles are opened so that they may be seen simply as a linear part of the backbone. The algorithm proceeds to walk down the carbon chain from the start to finish, and at each step, it counts the number of atoms of each element present in the carbon's neighborhood. The number of neighborhood levels considered depends on the user's input. For instance, first-level neighbors are atoms directly bonded to the original carbon, while *n*-th level neighbors are atoms connected to the original carbon in the graph by a shortest path of length *n*.

The algorithmic procedure involves converting the neighborhood information into a numerical representation or a counting array. This two-dimensional array is illustrated in Fig. 3. In this process, the columns of the array correspond to the carbons of the peptide, where the *n*-th column contains information about the *n*-th carbon in the chain. Rows in the array represent the unique elements in the element alphabet. For example, in the case where the element alphabet comprises hydrogen, carbon, nitrogen, oxygen, and sulfur, the counting array's first row records the number of hydrogen atoms sharing a bond with the corresponding carbon. The second row counts how many other carbons are adjacent to the given carbon atom. The third row indicates the count of nitrogen atoms which are immediate neighbors in the molecular graph, the fourth row displays the number of oxygen atoms, and the fifth row shows the number of sulfur atoms. Following one iteration through the entire alphabet, the information about second-level neighborhoods is recorded. For instance, the sixth row would contain the count of hydrogen atoms connected to the carbon atom by a shortest path of length two. This process continues for every element in the element alphabet and every level of neighborhood, up to the maximal level specified by the user. The result is a set of arrays, one for each compound in the data set, where the number of columns equals the length of the molecule's carbon chain, and the number of rows is equal to the product of the number of elements in the element alphabet and the maximum number of neighborhood levels chosen by the user. To facilitate easier data storage, these counting arrays are flattened column-by-column into single vectors and padded with zeroes, ensuring that all vectors in the data set have the same length for seamless processing by ML algorithms in subsequent tasks. For a given data set, these vectors are consolidated into a common data frame, where each row corresponds to the flattened counting array of the respective data points into a vector. The entire post-configuration procedure is also described in Algorithm 1.

**Algorithm 1:**
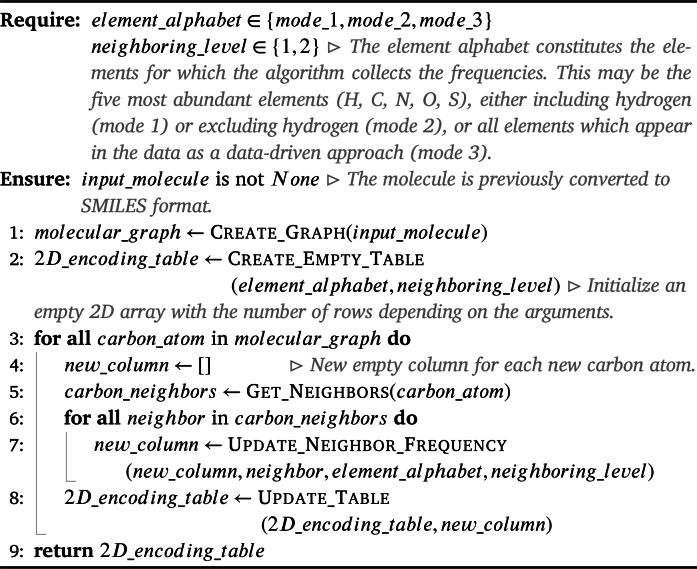
Post-configuration Procedure.

### 5.2 Interpretability

The original counting array for a selected peptide in the data set can be converted into a gray scale image. Initially, the array is normalized by dividing each cell by the highest entry in the array. Next, the cell entries are mapped to shades of gray with varying intensities, based on the cell's value, where 0 is represented by white and 1 is represented by black. The resulting array is then saved as a gray scale image, enabling the user to visually analyze samples of peptides and identify patterns.

Over the past years, there has been a push in the ML community to develop methods that use artificial intelligence in a more explicable way [24], [1]. More specifically, the method used to determine the importance of features when encoding molecular structures into machine readable input. First, it can help users debug their model, as understanding which features learners are basing their decisions on allows users to check whether they are relying on circumstantial artifacts that may be related to the label for the given data set, but not in the real world. Second, an understanding of the features that a model considers to be relevant for the prediction of certain properties can support our biological understanding of why certain compounds may exhibit these properties.

How feature importance can be calculated depends on the model that is used for the prediction task. In the case of Random Forest Classifiers, measures of feature importance for the entire data set such as decrease of impurity, permutation-based feature importance, or Shapley values [25], [26] can be calculated straightforwardly and are already included in the standard packages. If an interpretation of the feature importance for a specific data point is wanted or other models such as Deep Neural Networks, Support Vector Machines or k-Means Clustering are used, we may opt to use Layer-wise Relevance Propagation [27]. In the case of iCAN, they are computed as the average accumulation of the impurity decreases within each tree provided by the fitted attribute feature_importances_ in the RandomForestClassifier class.

Feature importance measures are mapped into the image space to create relevance heat maps. The heat maps visually display which parts of the molecule are relevant in the prediction of a certain property and may thus indicate that these parts are indeed relevant for the existence of said property in the compound. While relevance heat maps have been used in a study very recently [21], here the relevance of certain bits of the molecular fingerprints has been determined without linking this importance back to the molecular graph. To our knowledge, the potential for insight on a sequence-level has not been leveraged in molecular fingerprints to date.

## Code availability

The code is written in Python to guarantee high compatibility with existing methods in the bioinformatics and machine learning domains in general and molecular fingerprinting in specific. The code is available at https://github.com/ghattab/iCAN.

## Correspondence and requests

Should be addressed to Georges Hattab.

## CRediT authorship contribution statement

**Moritz Weckbecker:** Writing – original draft, Visualization, Software, Methodology, Investigation, Data curation. **Aleksandar Anžel:** Writing – review & editing, Visualization, Validation, Supervision, Software, Methodology, Investigation, Formal analysis. **Zewen Yang:** Writing – review & editing, Validation, Supervision, Software, Investigation, Formal analysis. **Georges Hattab:** Writing – review & editing, Validation, Supervision, Resources, Project administration, Methodology, Investigation, Funding acquisition, Conceptualization.

## Declaration of Competing Interest

The authors declare that they have no known competing financial interests or personal relationships that could have appeared to influence the work reported in this paper.

## Data Availability

All data sets are referenced and provided at https://github.com/ghattab/iCAN/tree/main/Data/Original_datasets.

## Appendix A Supplementary material

MMC iCAN_Interpretable_Molecular_Encodings_Supplemental. MMC
