# Supplementary material for: Interpretable molecular encodings and representations for machine learning tasks
Source: Comput Struct Biotechnol J. 2024 May 24;23:2326–36. doi: 10.1016/j.csbj.2024.05.035 (PMC11167246; doi:10.1016/j.csbj.2024.05.035)
Supplement: MMC — iCAN_Interpretable_Molecular_Encodings_Supplemental. [file mmc1.pdf]

Interpretable Molecular Encodings and Representations for Machine Learning Tasks

Moritz Weckbecker[a,1], Aleksandar Anžel[a,1], Zewen Yang[a,1], Georges Hattab[a,b]

[a] *Center for Artificial Intelligence in Public Health Research, (ZKI-PH), Robert Koch Institute, Nordufer 20, Berlin, 13353, Berlin, Germany*
[b] *Department of Mathematics and Computer science Freie Universität, Arnimallee 14, Berlin, 14195, Berlin, Germany*

Benchmarking Information

Benchmarking was performed using single-threading on a machine with the following properties:

- **OS**: Linux kernel: 5.4.0-153-generic
- **CPU**: AMD EPYC 7742 (16) @ 2.90GHz (Turbo 4.90GHz), Thread(s) per core: 2, Core(s) per socket: 8
- **Memory**: 16GB.

Data Set Properties

Each data set consists of two files. The first file represents peptides and/or proteins in `FASTA` or `SMILES` format. Each data set is additionally accompanied by a text file (named `classes.txt`) containing zeroes and ones separated by the new line character. Thus, the file contains a label vector where 1 indicates the presence and 0 the absence of the respective property for a specific data set.

Email addresses: `AnzelA@rki.de` (Aleksandar Anžel), `HattabG@rki.de` (Georges Hattab)

[1]These authors contributed equally to this work.

Preprint submitted to Computational and Structural Biotechnology Journal (CSBJ)May 22, 2024

Level-atom	C_0 neighborhood	C_1 neighborhood	C_2 ...
Level 1-H	quantity of H	quantity of H	quantity of H
Level 1-C	quantity of C	quantity of C	quantity of C
Level 1-O	quantity of O	quantity of O	quantity of O
Level 1-N	quantity of N	quantity of N	quantity of N
Level 1-S	quantity of S	quantity of S	quantity of S
Level 2-H	quantity of H	quantity of H	quantity of H
Level 2-C	quantity of C	quantity of C	quantity of C
Level 2-O	quantity of O	quantity of O	quantity of O
Level 2-N	quantity of N	quantity of N	quantity of N
Level 2-S	quantity of S	quantity of S	quantity of S

Table 1: Example encoding created with the iCAN method. The first encoding mode is used.

Data set	1st Encoding	1st F_1	2nd Encoding	2nd F_1	3rd Encoding	3rd F_1
ai4avp_2 *	iCAN_1	0.72	iCAN_2	0.71	iCAN_3	0.71
amp_fernandes	iCAN_3	0.82	dist_f	0.82	iCAN_1	0.81
amy_albase *	iCAN_2	0.93	iCAN_1	0.93	iCAN_3	0.93
amy_hex *	iCAN_1	0.69	iCAN_2	0.69	iCAN_3	0.68
c2pred *	iCAN_3	0.73	iCAN_2	0.72	iCAN_1	0.71
cpp_mixed	iCAN_1	0.89	iCAN_2	0.89	iCAN_3	0.89
cppsite2 *	iCAN_2	0.84	iCAN_1	0.83	iCAN_3	0.81
effectorp *	iCAN_1	0.57	iCAN_3	0.57	iCAN_2	0.56
foldamer_b *	iCAN_3	0.97	iCAN_1	0.97	iCAN_2	0.96
hiv_bevirimat	qsar	1.0	iCAN_2	0.79	cksaap	0.79
perm_cyc *	iCAN_2	0.85	iCAN_1	0.85	iCAN_3	0.85
sol_ecoli *	iCAN_1	0.72	iCAN_3	0.72	iCAN_2	0.71
toxinpred2 *	iCAN_1	0.75	iCAN_3	0.75	iCAN_2	0.75
toxinpred_swissprot *	iCAN_2	0.84	iCAN_1	0.83	iCAN_3	0.83
toxinpred_trembl *	iCAN_2	0.77	iCAN_1	0.76	iCAN_3	0.74

Table 2: Experiment 3. Overview of the top 3 encoding methods per data set. The list contains all data sets for which iCAN appears in one of the top 3 methods. The table is sorted alphabetically by the *Data set* column.
Data sets marked with an asterisk (*) could only be encoded using the iCAN method.

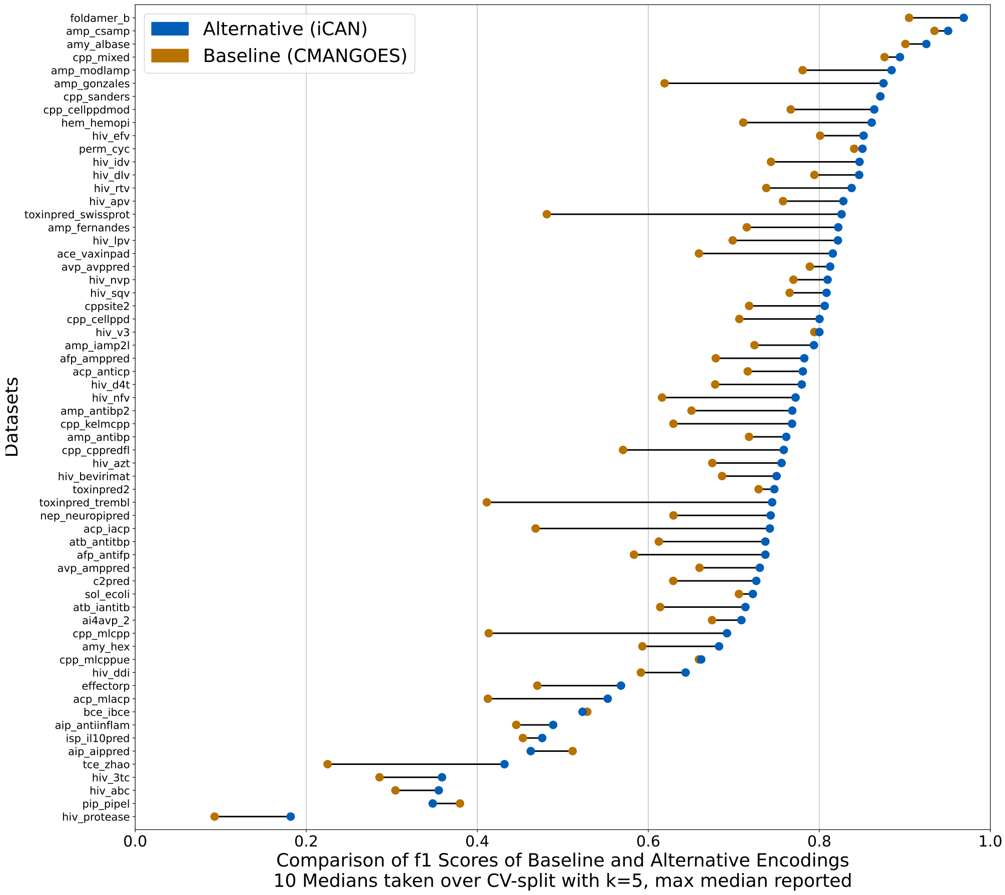

Figure 1: F_1-scores in classification task for CMANGOES (Baseline) and iCAN (Alternative) methods.

Data set	1st Encoding	1st F_1	2nd Encoding	2nd F_1	3rd Encoding	3rd F_1
ace__vaxinpad	cksaap	0.96	waac__a	0.94	dist__f	0.94
acp__anticp	apaac__	0.89	paac__l	0.89	waac__a	0.89
acp__iacp	paac__l	0.9	dist__f	0.9	qsorde	0.88
acp__mlacp	qsorde	0.76	waac__a	0.74	dist__f	0.74
afp__amppred	cksaap	0.91	dist__f	0.9	paac__l	0.9
afp__antifp	dist__f	0.9	cksaap	0.89	psekraac	0.88
aip__aippred	dde	0.62	egaac__	0.58	ctdd	0.58
aip__antiinflam	dde	0.67	waac__a	0.62	cksaap	0.62
amp__antibp	cksaap	0.96	qsorde	0.96	dist__f	0.95
amp__antibp2	qsorde	0.92	cksaap	0.92	apaac__	0.92
amp__csamp	qsorde	0.98	waac__a	0.98	cksaap	0.98
amp__gonzales	egaac__	0.89	cksaap	0.89	dpc	0.89
amp__iamp2l	dist__f	0.82	psekraac	0.8	ctdd	0.8
amp__modlamp	cksaap	0.93	dist__f	0.93	qsorde	0.92
atb__antitbp	cksaag	0.83	cksaap	0.83	dde	0.83
atb__iantitb	cksaap	0.8	dpc	0.8	apaac__	0.79
avp__amppred	cksaap	0.88	psekraac	0.87	apaac__	0.87
avp__avppred	cksaap	0.87	dpc	0.87	qsorde	0.86
bce__ibce	dde	0.65	ctdd	0.62	zscale	0.61
cpp__cellppd	dist__f	0.9	waac__a	0.9	aac	0.9
cpp__cellppdmod	dist__f	0.93	cksaap	0.93	waac__a	0.92
cpp__cppredfl	dist__f	0.91	waac__a	0.91	psekraac	0.91
cpp__kelmcpp	cksaap	0.85	dist__f	0.85	waac__a	0.84
cpp__mlcpp	cksaap	0.85	dist__f	0.84	psekraac	0.84
cpp__mlcppue	dist__f	0.7	psekraac	0.69	apaac__	0.69
cpp__sanders	dist__f	0.89	fldpc__	0.89	cksaag	0.89
hem__hemopi	apaac__	0.93	cksaap	0.92	qsorde	0.92
hiv__3tc	qsar	0.97	cksaap	0.9	psekraac	0.89
hiv__abc	qsar	0.97	cksaap	0.86	psekraac	0.85
hiv__apv	qsar	0.99	binary	0.9	cksaap	0.9
hiv__azt	qsar	0.98	cksaap	0.9	cgr__re	0.89
hiv__d4t	qsar	0.99	psekraac	0.88	binary	0.88
hiv__3i	qsar	0.98	cgr__re	0.8	binary	0.79
hiv__dlv	qsar	0.98	cksaap	0.92	dist__f	0.92
hiv__efv	qsar	0.98	cksaap	0.95	cgr__re	0.94
hiv__idv	qsar	0.99	binary	0.93	cksaap	0.93
hiv__lpv	qsar	0.99	dist__f	0.92	psekraac	0.92
hiv__nfv	qsar	0.99	cksaap	0.93	binary	0.92
hiv__nvp	qsar	0.98	cksaap	0.94	binary	0.93
hiv__protease	ngram__	0.79	aainde	0.53	blomap	0.46
hiv__rtv	qsar	0.99	binary	0.96	cksaap	0.96
hiv__sqv	qsar	0.99	binary	0.93	psekraac	0.92
hiv__v3	qsar	0.99	aainde	0.98	cksaap	0.98
isp__il10pred	dde	0.68	egaac__	0.62	fldpc__	0.61
nep__neuropipred	cksaap	0.88	dist__f	0.87	paac__l	0.87
pip__pipel	dde	0.56	zscale	0.5	flgc__a	0.49
tce__zhao	aainde	0.67	zscale	0.61	blomap	0.59

Table 3: Experiment 3. Overview of the top 3 encoding methods per data set (excluding iCAN). The list contains all data sets for which iCAN does not appear in any of the top 3 methods. The table is sorted alphabetically by the *Data set* column.

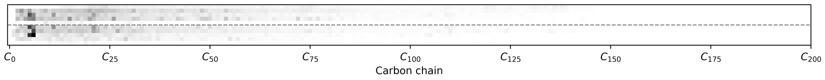

(a) Overview of the relevance heat map for the `ace_vaxinpad` data set.

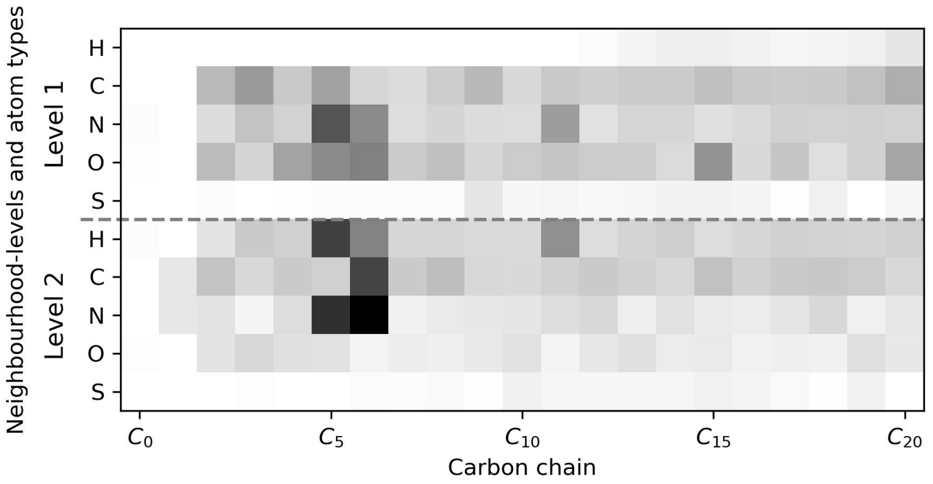

(b) Detailed view of the relevance heat map for the `ace_vaxinpad` data set.

Figure 2: Overview and detailed view of a relevance heat map for the `ace_vaxinpad` data set. iCAN is employed using the first mode to create the encodings, excluding the hydrogen atoms.
